# pVAX14DNA-mediated add-on immunotherapy combined with arsenic trioxide and all-*trans* retinoic acid targeted therapy effectively increases the survival of acute promyelocytic leukemia mice

**DOI:** 10.1038/bcj.2015.102

**Published:** 2015-12-11

**Authors:** S Patel, L Guerenne, P Gorombei, N Omidvar, M-H Schlageter, A A Alex, S Ganesan, R West, L Adès, V Mathews, P Krief, M Pla, P Fenaux, C Chomienne, R A Padua

**Affiliations:** 1 Université Paris Diderot, Institut Universitaire d'Hématologie, Unité Mixte de la Recherche de Santé (UMR-S) 1131, Paris, France; 2 Institut National de la Santé et de la Recherche Médicale (INSERM) Unité (U) 1131, Paris, France; 3 Haematology Department, Cardiff University School of Medicine, Cardiff, UK; 4 Assistance Publique Hôpitaux de Paris (AP-HP), Hôpital Saint Louis, Paris, France; 5 Department of Hematology, Christian Medical College and Hospital, Vellore, India; 6 Welsh Heart Research Institute, Cardiff University School of Medicine, Cardiff, UK

Novel immunotherapeutic approaches have recently highlighted the benefit of adjuvant add-on immunotherapy approaches combined with chemotherapy or targeted therapies. To validate this approach, we took advantage of a mouse transplantable acute promyelocytic leukemia (APL) model^1^ that has proven to be a very robust and reproducible *in viv*o pre-clinical model of all the current and prospective therapeutic approaches of this leukemia as a proof-on concept for other malignancies.^2^ Of relevance to this study, this model was used to demonstrate a synergy of all-*trans* retinoic acid (ATRA) and arsenic trioxide (ATO) for APL eradication and elucidate its mechanism of action,^3^ and this strategy is used as targeted therapies for patients.^4, 5, 6^

Using this preclinical model, we previously demonstrated the efficacy of combined fusion gene promyelocytic leukemia-retonoic acid receptor alpha (PML-RARA) DNA administration with ATRA on mouse survival and leukemia-initiating cell eradication.^7, 8, 9, 10, 11^ We have recently reported the similar efficacy of a novel nonspecific DNA construct pVAX14 with adjuvant properties and with an equally effective impact on the survival of these APL mice in combination with ATRA.^8^

More recently, ATRA combined with ATO has become the reference treatment for newly diagnosed APL.^4^ Thus, this present study focused on the therapeutic potential of pVAX14 to increase the efficacy of the combination ATO+ATRA treatment (schematic diagram of the protocol described in Figure 1a). Of note, to detect the increased efficacy a suboptimal dose of ATRA (5 mg) was used. All animal procedures complied with national and international guidelines and this study was approved by the local ethical committee (Committee on the Ethics of Animal Experiments—Paris Nord C2EA-121, approval no. 2014-IUH006). Survival of the APL mice treated with pVAX14+ATO+ATRA was significantly (*P*<0.03) superior to that of the mice treated with Vehicle+ATO+ATRA (Figure 1a). A survival of more than 300 days was observed in 92% of the mice treated with pVAX14+ATRA+ATO compared with 58% of the mice treated with Vehicle+ATO+ATRA. We have already reported that APL mice treated with pVAX14 alone showed little benefit in terms of survival;^7^ Vehicle+ATO-, Vehicle+ATRA- and pVAX14+ATO-treated mice relapsed and died (Supplementary Figure S1a and Supplementary Table S1) with no improvement in peripheral blood (PB) platelet counts (Supplementary Figure S1b and Supplementary Table S2). Both pVAX14+ATO+ATRA and Vehicle+ATO+ATRA treatment groups demonstrated rescue of PB platelet counts compared with placebo (*P*<0.0005, Supplementary Figure S1b and Supplementary Table S2). We have previously shown that PB platelet counts correlated well with the disease.^10^

Monitoring of PB cells by RT-qPCR analysis of *PML-RARA* offers a measurable evaluation of disease control during the treatment in this model. The primers used are detailed in Supplementary Table S3. A significant (*P*<0.04) difference was observed between the treatment groups with (*n*=17) or without pVAX14 (*n*=17) (Figure 1b inset). The difference in minimal residual disease (MRD) between the pVAX14 and Vehicle treatment groups and placebo was significant (*P*<0.01, Figure 1b).

To further monitor the efficacy of pVAX14 with ATRA+ATO, a biomarker we have previously described, MyD88 transcript levels were investigated using primer sequences detailed in Supplementary Table S3.^8, 12^ MyD88 is an adaptor protein that mediates numerous biologically important signal transduction pathways including pathways involved in DNA's mechanism of action.^12^ Transcript levels of *MyD88* were significantly (*P*<0.017) higher in APL mice treated with pVAX14+ATO+ATRA (*n*=15) compared with mice treated with Vehicle+ATO+ATRA only (*n*=20) (Figure 1c).

The involvement of humoral and cellular immune responses was then investigated (Figure 2). Western blot analysis done on sera from mice treated with ATO+ATRA+pVAX14 showed presence of antibodies recognizing human RARA (Figure 2a inset). To measure the levels of anti-RARA antibodies, the sera of two experimental groups of APL mice treated with pVAX14+ATO+ATRA (*n*=4 mice) and Vehicle+ATO+ATRA (*n*=5 mice) were assessed with an enzyme-linked immunosorbent assay (ELISA) using the GST and GST-RARA, we have previously described.^9, 11^ The levels of anti-RARA antibodies corroborated the Western blot analysis as higher levels were predominantly observed (*P*<0.04) in mice treated with pVAX14+ATO+ATRA (Figure 2a).

The involvement of T cells in the anti-leukemic effect elicited by pVAX14+ATO+ATRA therapy was also noted, in the percentage of memory T cells (memT), which was significantly (*P*<0.04) higher in pVAX14+ATO+ATRA-treated APL (*n*=11) mice than in control mice (*n*=9) (Figure 2b), as was the production of IFNγ by specific T cells, which was only increased in the pVAX14 treatment group (*n*=14) compared with the Vehicle treated mice (*n*=10) (*P*<0.001, Figure 2c). Two functional assays were used to evaluate the cytotoxic activity of T cells against precursor bone marrow (BM) cells. First we applied the assay described by Quintarelli *et al.*^13^ and that we have previously used,^8^ in which APL BM targets were cocultured with CD3+ spleen cells from either Vehicle+ATO+ATRA- (*n*=4) or pVAX14+ATO+ATRA- (*n*=5) treated APL mice. As shown in Figure 2d, the incubation of CD3+ spleen cells from pVAX14+ATO+ATRA mice with APL precursors leads to a significant (*P*<0.02) reduction of colony-forming units (CFUs). These effectors had no effect on the growth of normal friend leukemia virus, strain B from the National Institutes of Health (NIH) (FVB/N) BM CFUs (Supplementary Figure S2). In a second assay, specific APL BM precursor killing was determined by a carboxyfluorescein diacetate succinimidyl ester (CFSE)-based method as previously described.^7, 14^ In this assay, effectors were the splenocytes from Vehicle+ATO+ATRA- (*n*=3) and pVAX14+ATO+ATRA- (*n*=3) treated mice and target cells were the BM from APL diseased mice, assayed in triplicate. Effectors from pVAX14+ATO+ATRA-treated mice show a more efficient elimination of target cells (51% compared with 65%) at an effector:target ratio of 100:1 (*P*<0.05) (Figure 2e and Supplementary Figure S3).

These studies show that the add-on therapy of pVAX14 DNA results in increased survival efficacy with strong molecular responses when added to ATO and ATRA treatment. Biomarkers of response showed an increase in *MyD88* transcripts, suggesting the activation of the signaling pathway to NF-κB and induction of innate immune responses. Humoral responses were detected with higher levels of anti-RARA antibodies in the pVAX14-treated group. As expected of a DNA-mediated response, memory T cells and IFNγ-producing cells were increased. These changes translated into enhanced cytotoxic T-cell responses with the inhibition of APL BM progenitor growth and cytotoxic T-lymphocytes targeting APL cells. We hypothesize that the pVAX14 sequences, which we have demonstrated to induce immune responses,^8^ potentiate the immune responses already initiated by the release of the PML-RARA tumor antigen due to terminal differentiation and immunogenic cell death^15^ induced by ATRA and ATO. An add-on adjuvant immunotherapy approach may have utility in preventing relapse and could be considered for the rare high-risk APL patients who fail to respond to ATO+ATRA therapy. As pVAX14 is acting as a nonspecific DNA adjuvant with the specificity coming from the tumor cells themselves, this immunotherapeutic strategy may be applicable to other malignancies. We have already demonstrated this to be the case in our preclinical model of high-risk MDS^8^ and we predict that this approach will be applicable to solid tumors in combination with agents that induce immunogenic cell death to enable tumor antigen shedding/spreading and initiation of immune responses, which pVAX14 then potentiates.

## Figures and Tables

**Figure 1:**
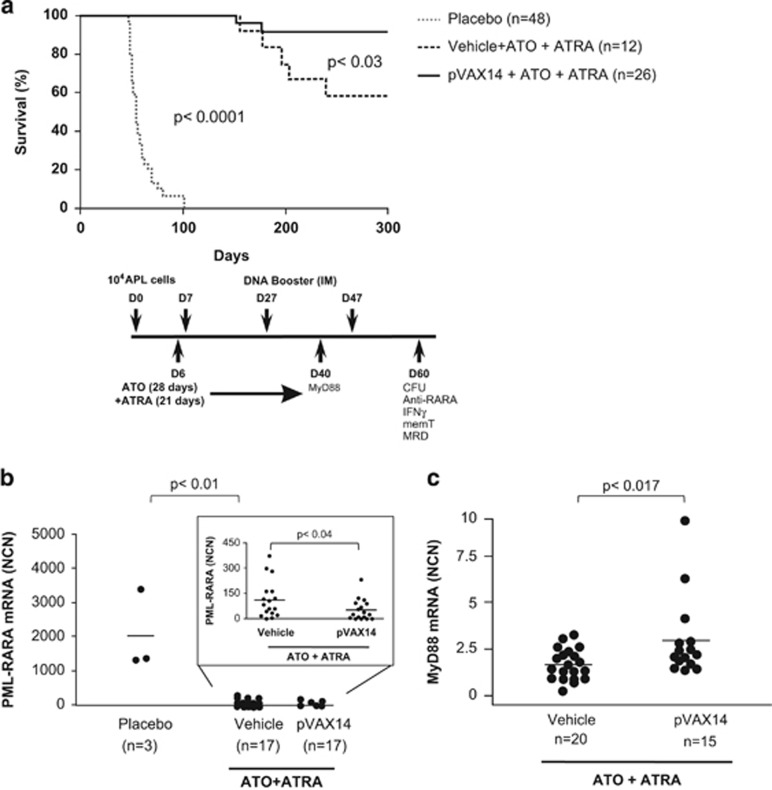
Increased survival of APL mice by treatment with pVAX14 in addition to ATO and ATRA. (**a**) Kaplan–Meier survival curves showing increased survival in the APL mice; pVAX14+ATO+ATRA showed the best survival with significant difference compared with Vehicle+ATO+ATRA (*P*<0.03). The differences between pVAX14+ATO+ATRA or Vehicle+ATO+ATRA and Placebo were significant (*P*<0.0001); the schematic diagram of the protocol used is illustrated. APL blast cells from the spleen (10^4^) were injected intravenously on day 0 (D0), followed by ATRA (5-mg–21-day release pellet, Innovative Research of America, Sarasota, FL, USA) on day 6 (D6). Vehicle (Hepes buffered saline solution) or pVAX14 DNA (2 × 50 μg) was administered intramuscularly on day 7 (D7) and every 20 days for a total of 3 cycles. ATO was prepared (Sigma Chemical Co, St Louis, MO, USA)^3^ and administered intraperitoneally daily at the concentration of 5 μg/g/mice for 28 consecutive days starting on D6. (**b**) Minimal residual disease (MRD) in APL mice treated with pVAX14+ATO+ATRA. Primer sequences are detailed in Supplementary Table S3. Results were expressed as normalized copy numbers (NCN) of *PML-RARA* transcripts using *Abl* as a housekeeping gene.^8, 10^ A significant reduction in MRD was observed on day 60 (D60) of pVAX14+ATO+ATRA-treated APL mice compared with Vehicle+ATO+ATRA (*P*<0.04) mice (inset); pVAX14+ATO+ATRA or Vehicle+ATO+ATRA versus placebo were significantly different (*P*<0.01). (**c**) Detection of significantly increased *MyD88* expression on day 40 (D40) of pVAX14+ATO+ATRA-treated APL mice compared with Vehicle+ATO+ATRA-treated mice (*P*<0.017). Primer sequences are shown in Supplementary Table S3. The nonparametric, unpaired, two-tailed, Mann–Whitney test was used to compare different groups using the Prism software.

**Figure 2:**
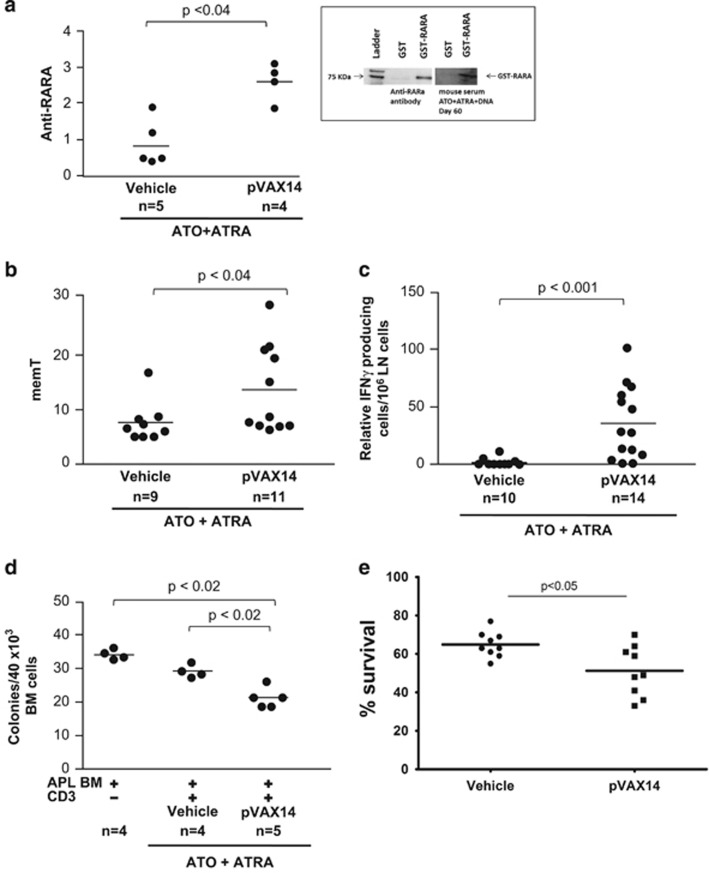
Biomarkers of pVAX14 efficacy in APL in addition to ATO and ATRA. (**a**) ELISAs were undertaken as described previously,^9, 11^ showing increased anti-RARA antibody production in pVAX14+ATO+ATRA-treated APL mice compared with Vehicle+ATO+ATRA-treated mice on day 60 (*P*<0.04). The Y-axis represents anti-RARA production expressed as follows: the ratio of specific absorbance (SA)/anti-RARA antibody 9alpha was calculated and this ratio was then divided by the median ratio obtained in control mice (ATRA alone); the specificity of the anti-RARA antibody response was confirmed by western blots (inset) using recombinant GST or GST-RARA proteins as described previously^9^ with either anti-RARA antibody or serum from day 60 of mice treated with pVAX14+ATO+ATRA. (**b**) Determination of memory T cells (memT cells), percentage of CD44^hi^/CD62L^lo^population within the CD4+, as a measure of memT cells was undertaken on PB of the mice treated, showing a significant increase in pVAX14+ATO+ATRA-treated mice compared with Vehicle+ATO+ATRA-treated APL mice on day 60 (*P*<0.04). (**c**) Lymph node cells from pVAX14+ATO+ATRA-treated mice showed a significant increase in interferon-γ (IFNγ)-secreting cells compared with those originated from Vehicle+ATO+ATRA-treated mice at day 60 using the Mann–Whitney one-tailed analysis (*P*<0.001). The T cells were stimulated with irradiated APL cells and the numbers of unstimulated IFNγ-producing cells were subtracted from the stimulated values. (**d**) Inhibition of APL colony-forming units (CFU) by CD3+ enriched cells was measured as described.^8, 13^ Fresh bone marrow (BM) APL cells from a mouse with a high leukemic blast count were used as targets. CD3+ enriched spleen cells of treated APL mice on day 60 using magnetic beads from Miltenyi Biotec (Bergisch Gladbach, Germany) from APL-treated mice were co-cultured in a methylcellulose cell culture assay (Methocult, Stem Cell Technologies, Vancouver, Canada) with BM cells from normal friend leukemia virus, strain B from the National Institutes of Health (NIH) (FVB/N) or APL diseased mice. The ratio of BM to CD3+ was 1:10. CFU colonies were counted at day 7 of incubation. CD3+ cells from pVax14+ATO+ATRA-treated mice significantly reduced APL CFU compared with CD3+ cells from Vehicle+ATO+ATRA-treated mice (*P*<0.02). Unless stated, nonparametric, unpaired, two-tailed, Mann–Whitney test was used to compare different groups using the Prism software. (**e**) Increased cytotoxic cells in pVAX14-treated mice. A cytotoxic carboxyfluorescein diacetate succinimidyl ester-based assay was performed as previously described.^14^ Effector spleen cells from each cohort was restimulated using irradiated APL cells for 4 days at 37 °C. 10^4^ CFSE-labelled APL BM targets were incubated at the following effector:target ratios: 0:1, 25:1, 50:1 and 100:1 (Supplementary Figure S3) for 6 h at 37 °C in 200 μl of culture medium (RPMI); the percentage survival was calculated as follows: % survival (y-axis)=(absolute count of viable CFSE+PI-targets with effector (*t*=6 h))/(absolute count of viable CFSE+PI-targets only (*t*=6 h))x100. Effector cells of pVAX14+ATO+ATRA-treated mice have increased cytotoxicity against APL cells compared with effectors from Vehicle+ATO+ATRA-treated mice at an E:T of 100:1 (*P*<0.05); *n*=3 mice were assayed in triplicate. A two-tailed unpaired *t*-test statistical analysis was used.
