# Supplementary material for: pVAX14DNA-mediated add-on immunotherapy combined with arsenic trioxide and all-trans retinoic acid targeted therapy effectively increases the survival of acute promyelocytic leukemia mice
Source: Blood Cancer J. 2015 Dec 11;5(12):e374–. doi: 10.1038/bcj.2015.102 (PMC4735069; doi:10.1038/bcj.2015.102)
Supplement: Supplementary Information [file bcj2015102x1.pdf]

Supplementary Materials for

pVAX14DNA-mediated add-on immunotherapy combined with arsenic trioxide and all-trans retinoic acid (ATO+ATRA) targeted therapy effectively increases survival of APL mice

Satyananda Patel, Laura Guerenne, Petra Gorombei, Nader Omidvar, Marie-Helene Schlageter, Ansu Abu Alex, Saravanan Ganesan, Robert West, Lionel Adès, Vikram Mathews, Patricia Krief, Marika Pla, Pierre Fenaux, Christine Chomienne, Rose Ann Padua*

*To whom correspondence should be addressed. E-mail: rose-ann.padua@inserm.fr

This pdf includes:

Table S1 – S3

Figure S1- S3

Table S1. Statistical analyses of survival curves on Figures 1a and S1a

Groups	log-rank (Mantel-Cox)		Gehan-Breslow-Wilcoxon	
	Chi-2 value	p value	Chi-2 value	p value
pVAX14+ATO+ATRA vs Vehicle+ATO+ATRA	4.06	P<0.02	4.4	P<0.03
pVAX14+ATO+ATRA vs pVAX14+ATO	48.71	P<0.0001	43.36	P<0.0001
pVAX14+ATO+ATRA vs ATRA	59.49	P<0.0001	44.36	P<0.0001
pVAX14+ATO+ATRA vs ATO	60.78	P<0.0001	46.53	P<0.0001
pVAX14+ATO+ATRA vs Placebo	57.22	P<0.0001	43.16	P<0.0001
Vehicle+ATO+ATRA vs pVAX14+ATO	26.46	P<0.0001	22.03	P<0.0001
Vehicle+ATO+ATRA vs ATRA	27.96	P<0.0001	20.72	P<0.0001
Vehicle+ATO+ATRA vs ATO	34.55	P<0.0001	23.57	P<0.0001
Vehicle+ATO+ATRA vs Placebo	32.71	P<0.0001	21.99	P<0.0001
ATRA vs pVAX14+ATO	6.12	P<0.01	4.73	P<0.03
ATRA vs ATO	22.1	P<0.0001	17.6	P<0.0001
ATRA vs Placebo	74.32	P<0.0001	68.78	P<0.0001
ATO vs pVAX14+ATO	0.61	P<0.44	0.2	P<0.65
ATO vs Placebo	43.28	P<0.0001	46.42	P<0.0001
pVAX14+ATO vs Placebo	20.09	P<0.0001	17.58	P<0.0001

Table S2. Statistical analyses of peripheral blood platelet counts on Figure S1b

Groups	Mann-Whitney test (P value)
	Platelets
Placebo vs ATRA	0.0004
Placebo vs ATO	0.0058
Placebo vs ATO + ATRA	0.0005
Placebo vs ATO + pVAX14	0.6
Placebo vs ATRA + ATO + pVAX14	0.0002
ATRA vs ATO	0.1524
ATRA vs ATO + ATRA	0.6096
ATRA vs ATO + pVAX14	0.0017
ATRA vs ATRA + ATO + pVAX14	0.4046
ATO vs ATO + ATRA	0.4021
ATO vs ATO + pVAX14	0.02
ATO vs ATRA + ATO + pVAX14	0.0423
ATRA +ATO vs ATO + pVAX14	0.0013
ATRA +ATO vs ATRA + ATO + pVAX14	0.3353
ATO + pVAX14 vs ATRA + ATO + pVAX14	0.0012

Table S3. Primer sequences for *Abl*, *PML-RARA* and *MyD88*

Primer	Sequence (5'-3')	Amplicon (bp)
mAbl-F	GAAGACCTTGAAGGAGGACACCATG	183
mAbl-R	GGGTACACACCCCTAGCAGCT	
PMLRARA-F	GTCTTCCTGCCCAACAGCAACC	190
PMLRARA-R	CTCACAGGCGCTGACCCCATAGT	
MyD88-F	CGCGCATCGAGGAGGACTGC	156
MyD88-R	CCGGCGTTTGTCCTAGGGGGT	

Figure S1

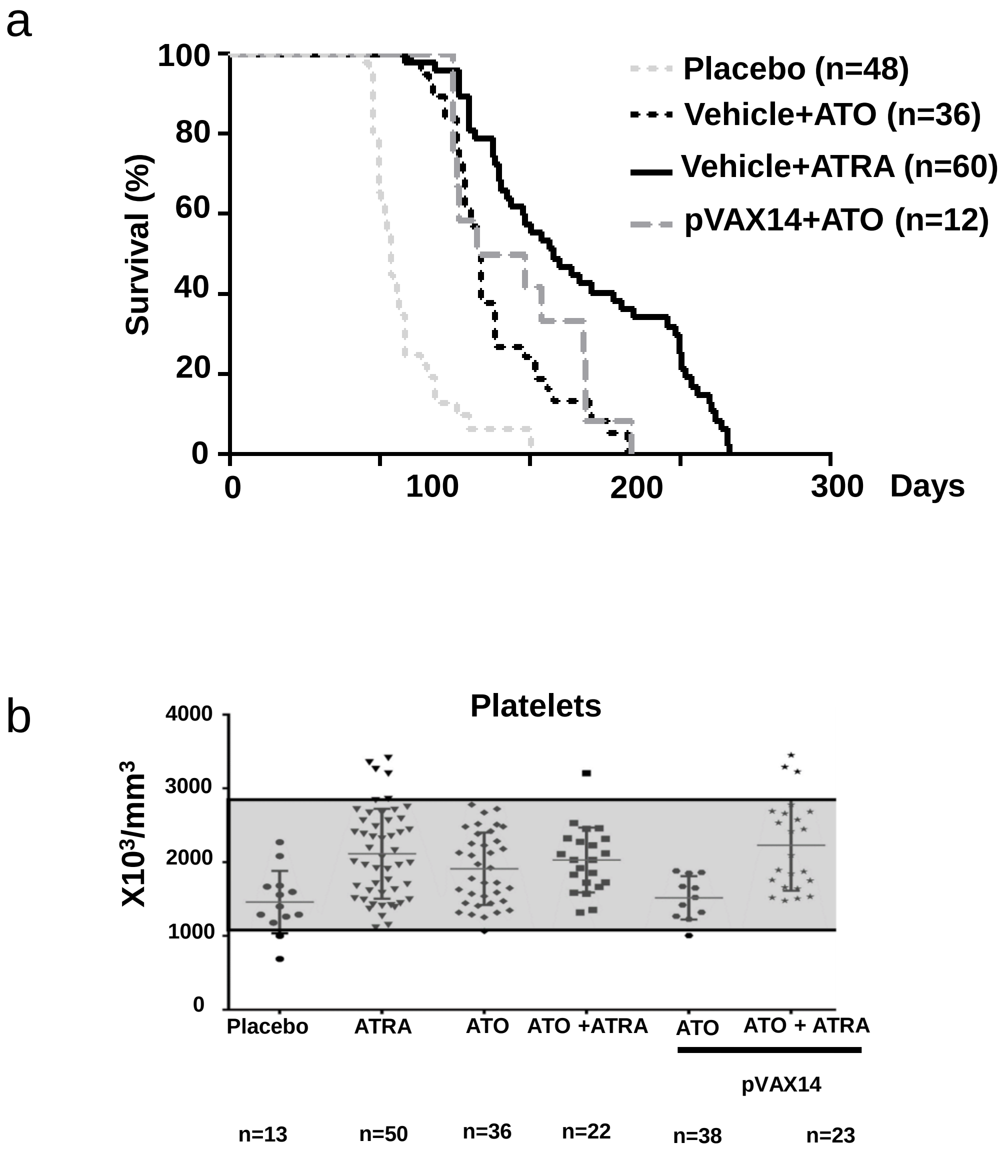

Figure S1. Kaplan-Meier survival curves and platelet counts of APL mice.

a. Kaplan- Meier survival curves of placebo [injected with phosphate buffered saline (PBS)] or Vehicle (Hepes buffered saline solution) +ATO, Vehicle+ATRA and pVAX14+ATO-treated APL mice showing that all mice relapse and die. Statistical analyses are on Supplementary Table S1.
b. Peripheral blood (PB) counts of APL mice injected with Placebo (PBS), Vehicle+ATRA (ATRA), Vehicle+ATO (ATO), Vehicle+ATO+ATRA (ATO+ATRA), pVAX14+ATO or VAX14+ATO+ATRA on day 60 of protocol illustrated on Figure 1a. The normal range is delineated in grey; statistical analyses are on Table S2, nonparametric, unpaired, two tailed, Mann-Whitney test was used to compare different groups. The Prism software was used for the t-test analysis.

Figure S2

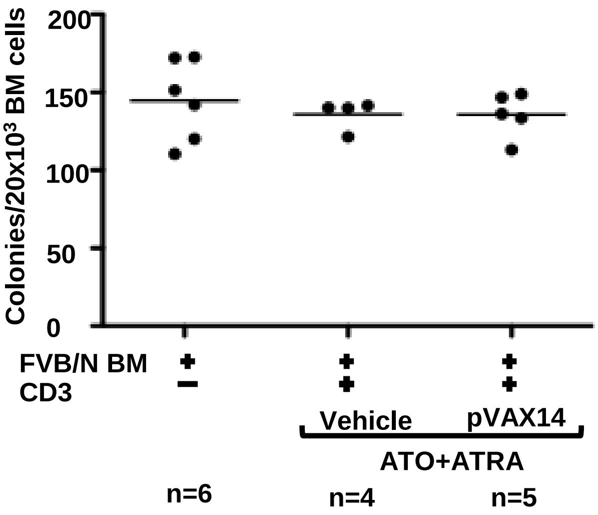

Figure S2. No adverse effects in normal myeloid progenitors.
CD3+ effectors from APL mice treated as shown showing no effect on FVB/N progenitors plated at an effector:target (E:T) of 10:1 using methods detailed in legend to Figure 2d.

Figure S3

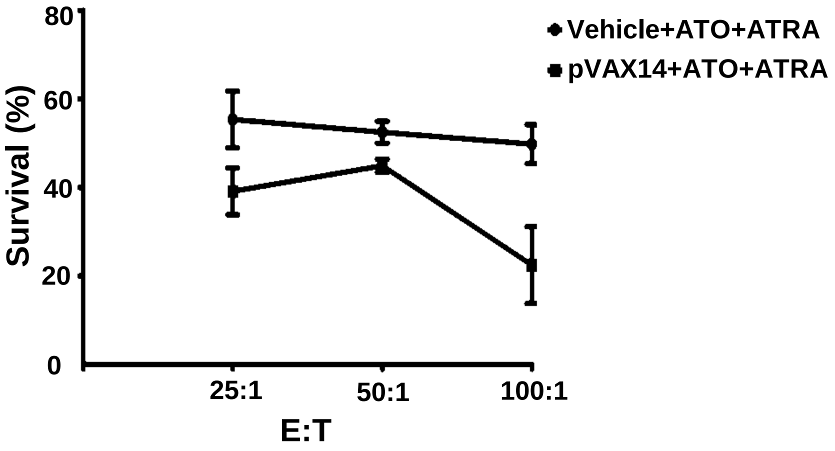

Figure S3. Increased cytotoxic cells in pVAX14-treated mice.
A cytotoxic CFSE-based assay was performed as previously described (14) and detailed in legend to Figure 2e. Spleen cells from each cohort was re-stimulated using irradiated APL cells for 4 days at 37°C. 10^4 CFSE$^-$labelled APL bone marrow (BM) targets were incubated at the following E:T ratio: 25:1, 50:1, and 100:1. Effector cells of pVAX14+ATO+ATRA-treated mice have increased cytotoxicity against APL cells compared to effectors from Vehicle+ATO+ATRA-treated mice at an E:T of 100:1 ($p<0.05$) and at all the three ratios; n=3 mice were assayed in triplicate. A 2-tailed unpaired t-test statistical analysis was used.
